# RAS signaling and immune cells: a sinister crosstalk in the tumor microenvironment

**DOI:** 10.1186/s12967-023-04486-9

**Published:** 2023-09-05

**Authors:** Yongting Liu, Bin Xie, Qiong Chen

**Affiliations:** 1 Department of Geriatrics, Respiratory Medicine, Xiangya Hospital, Central South University, Changsha, 410008 China; 2 National Clinical Research Center for Geriatric Disorders, Xiangya Hospital, Central South University, Changsha, 410008 China; 3 Xiangya Lung Cancer Center, Xiangya Hospital, Central South University, Changsha, 410008 China

**Keywords:** RAS mutation, Immune cell infiltration, Immune escape, Cancer vaccine, Adoptive cell therapy

## Abstract

The rat sarcoma virus (RAS) gene is the most commonly mutated oncogene in cancer, with about 19% of cancer patients carrying RAS mutations. Studies on the interaction between RAS mutation and tumor immune microenvironment (TIM) have been flourishing in recent years. More and more evidence has proved that RAS signals regulate immune cells' recruitment, activation, and differentiation while assisting tumor cells to evade immune surveillance. This review concluded the direct and indirect treatment strategies for RAS mutations. In addition, we updated the underlying mechanisms by which RAS signaling modulated immune infiltration and immune escape. Finally, we discussed advances in RAS-targeted immunotherapies, including cancer vaccines and adoptive cell therapies, with a particular focus on combination strategies with personalized therapy and great potential to achieve lasting clinical benefits.

## Introduction

Cancer is daunting because of its diversity and complexity. Genomic instability and mutation are considered to be important components in the acquisition of malignant traits [1]. The rat sarcoma virus (RAS) gene is the most commonly mutated oncogene in cancers, with about 19% of cancer patients carrying the RAS mutation [2]. The RAS family has long been the most notorious of the undruggable targets. Since The key discovery of cysteine-binding pockets in the KRAS G12C protein, the past decade has witnessed an astonishing outpouring of research on RAS inhibitors. The evidence is increasingly compelling that RAS oncogenic signaling extends beyond cancer cells to orchestrate the microenvironment. Research on the intersections between RAS mutations and tumor immune microenvironment (TIM) has blossomed, producing abundant demonstrations that RAS signals functionally regulate the recruitment, activation, and differentiation of immune cells, and coordinate tumor cells to evade immune surveillance [3].

Together, we reviewed the direct and indirect treatment strategies for RAS mutations and the attendant resistance challenges. In addition, we updated the underlying mechanisms by which RAS signaling modulated the TIM and discussed advances in RAS-targeted immunotherapies, including cancer vaccines and adoptive cell therapies. Finally, we look forward to the future of RAS mutation cancer treatment, with a particular focus on personalized therapy and combination strategies to achieve lasting clinical benefits.

## RAS signaling in cancers

The human RAS gene family includes KRAS, HRAS and NRAS, all of which encode 21 kDa small GTP-enzyme proteins. RAS protein product consists of G-domain at the N-terminal and hypervariable region (HVR) at the C-terminal. The G domain is highly conserved and consists of the P-loop, switch I, and switch II regions. By combining guanine exchange factors (GEFs) and GTPase activating proteins (GAPs), this domain acts as a switch between the activation and inactivation of GDP-GTP exchange. HVR is required for RAS protein anchoring to the plasma membrane to function [4, 5].

**Fig. 1:**
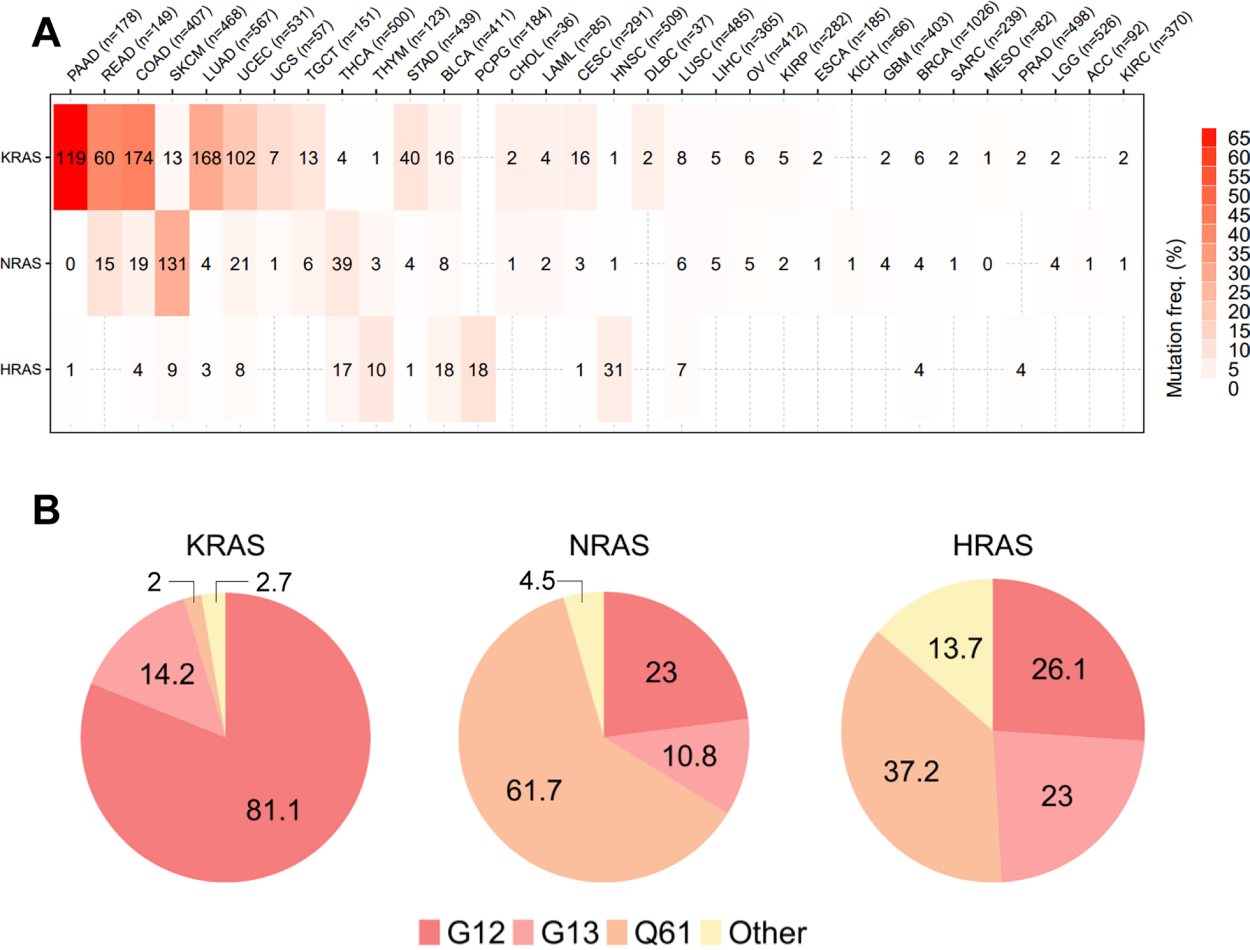
Overview of RAS mutation data. **A** The proportion of RAS mutation hotspots from TCGA database. **B** G12, G13, and Q61 occupy over 85% of all RAS mutations according to COSMIC database

Although RAS proteins exhibit some structural homology and share similar functional and biochemical properties, the oncogenic potential of each RAS isoform varies by the tissue, codon, and mutation frequency. RAS mutations appear at different rates in various malignancies. Pancreatic cancer has the highest incidence (> 85%), followed by colorectal cancer (~ 40%) and NSCLC (~ 30%). Five mutations (G12D, G12V, G12C, G13D and Q61R) accounted for 70% of all patients with Ras mutations [6–8]. KRAS is by far the most frequently mutated Ras isoform. The study of NRAS mutation mainly focuses on skin melanoma [9]. HRAS mutation drives recurrence and metastasis of head and neck squamous cell carcinoma [10, 11] (Fig. 1).

KRAS mutations lead to uncontrolled activation of the MAP kinase pathway, PI3K-AKT-mTOR pathway, and RAS-related protein (RAL)-NF-KB pathways [12]. Growing evidence supports the proposition that mutated KRAS play a carcinogenic role by increasing tumor blood vessels, tumor invasiveness, matrix remodeling and immunosuppression. In addition to the cumulative evidence for the regulation of TME by mutated KRAS, the equivalent studies describing such effects of carcinogenic NRAS and HRAS are limited, so their effects on TME have not been studied abundantly.

## Immune cells infiltration under RAS mutation

### Tumor-associated macrophage

Under the action of different stimulators, tumor-associated macrophages (TAMs) can be polarized in different directions: anti-tumor phenotype (M1) and pro-tumor phenotype (M2), thus playing a “double-edged sword” role in the occurrence and development of tumors. M1 macrophages are recruited mainly in the early stage of cancer occurance, causing a persistent chronic inflammatory microenvironment and facilitating the initiation of cancer. However, during tumor progression, TAMs undergo metabolic reprogramming to the M2 type, triggering immunosuppressive pro-tumor signals [13, 14].

KRAS G12D mutation in pancreatic acinar cells has been found to induce the expression of (ICAM-1), which triggered the recruitment of M1-like macrophages. Subsequently, the M1 macrophages secreted TNF and matrix metalloproteinase-9 (MMP-9), which cooperated with KRAS signaling to accelerate the pathogenesis of pancreatic cancer [15, 16]. Dai et al. further identified KRAS G12D as a key mediator in the communication between cancer cells and macrophages. They found that pancreatic cancer cells could release exosomes containing the KRAS G12D protein, which were taken up by macrophages through an AGER-dependent mechanism. KRAS G12D induced macrophages to switch to a pro-tumor M2 phenotype through STAT3-dependent fatty acid oxidation [17].

The example in CRC also showed that KRAS mutations (regardless of mutation type) reprogram macrophages, manifesting an increase in M2 phenotype with high CD206/low HLA-DR. These changes were attributed to the combined effect of CSF2 and lactic acid produced by HIF-1α signal transduction in tumor cells. Mutant KRAS stabilized HIF-1α by increasing the production of reactive oxygen species [18].

### Neutrophil

As a critical component of innate immune defense, the indispensable role of neutrophils in TIM has been gradually recognized [19]. Observations in NSCLC supported the proposition that oncogenic KRAS induced IL-8 overexpression. Meanwhile, MEK inhibitors significantly decreased IL-8 expression, while p38 inhibitors increased IL-8 expression [20]. It has been reported that IL-8 can directly induce the production of the neutrophil extracellular traps (NETs). A novel and predominantly pro-tumorigenic role of NETs in cancer is emerging [21, 22]. Interestingly, mutated KRAS proteins have recently been reported to be involved in cell communication [23]. After injection of APC-KRAS G12D-derived exosomes, upregulation of IL-8 was observed in peripheral blood, spleen, and mesenteric lymph nodes of APC-WT mice, as well as neutrophil infiltration and NETs formation. Secondly, cell-derived exosomes could adhere to NETs under static conditions in vitro. The research presented above suggested that exosomes might transfer mutated KRAS to recipient cells and trigger IL-8 production and recruitment of neutrophils, ultimately leading to the deterioration of CRC.

In addition, Esra et al. generated bitransgenic mice expressing a conditional IL-17A allele along with conditional KRAS G12D and demonstrated the role of IL-17 in KRAS mutant lung tumors. IL17 is mainly produced by Th17 cells, CD8 T cells, and γδT cells in the tumor microenvironment. High levels of IL17A in KRAS mutant mice promoted IL-6 and G-CSF secretion by binding to IL-17 receptor A on the surface of lung cancer cells, leading to increased invasion of tumor-associated neutrophils [24, 25]. Analysis in the TCGA database also prompted that KRAS mutated lung tumors exhibited significantly higher circulating IL-17A, but the mechanism remained to be elucidated [26].

Co-mutant signals also seem to contribute to the infiltration of immune cells in RAS mutant tumors. STK11/LKB1 inactivation is common in KRAS-mutated lung cancer. The study found that KRAS-LKB1 mutant lung cancer silenced the STING pathway owing to intrinsic mitochondrial dysfunction, resulting in a more aggressive and metastatic phenotype, significantly reduced survival rate, and drug sensitivity [27, 28]. Meanwhile, higher levels of neutrophils were observed in KRAS-LKB1 mutated lung cancer compared to KRAS alone, as well as many chemokines such as CXCL7, CXCL3, and CXCL5, all of which act through CXCR2 on neutrophils [29].

### Regulatory T cell

Regulatory T cells (Tregs) are an immunosuppressive subgroup of CD4+ T cells. Tregs not only directly kill cancer cells by secreting granulozyme and perforin, but also competitively consume IL-2 with effectors T cells and simultaneously produce TGF-β, IL-10 and IL-35, leading to immunosuppression [30].

In the lung cancer model, KRAS G12V induced tumor cells to secrete IL-10 and TGF-β1 by activating the MEK-ERK-AP1 pathway to induce Tregs infiltration. In this context, a high proportion of CD4+CD25-T cells were transformed into Tregs, which were characterized by the high expression of FOXP3, CTLA-4, and CD122. Even before tumor occurrence, KRAS inhibition reduced the number of Treg induced by tobacco carcinogen NNK in lung tissue [31]. Mechanically, KRAS signals further assist Tregs to promote GATA3/NOS2-related immunosuppression through the STING/ILC2 axis, thereby increasing lung metastatic load [32, 33]. Similar results were seen in CRC and breast cancer.

Liu et al. combined the sequencing data and TCGA analysis of CRC patients and found that Tregs increased in KRAS mutant CRC, while macrophage M1 and activated CD4 memory T cells decreased [34]. Curcumin, an inhibitor of the MEK/ERK signal, can inhibit the production of TGF-β in tumor cells, thus reducing Treg infiltration in breast cancer [35].

### Myeloid-derived suppressor cell

Myeloid-derived suppressor cells (MDSCs), are a group of myeloid-derived suppressor cells. MDSCs are precursors of dendritic cells, macrophages and granulocytes, and have the ability to negatively regulate immune response [36]. Liao et al. summarized the KRAS-IRF2-CXCL3-CXCR2-MDSC axis to clarify the regulatory mechanism of KRAS on tumor immunity in CRC [37]. Basically, KRAS G12D inhibited the expression of interferon regulatory factor 2 (IRF2), leading to the upregulation of CXCL3, and then recruitment of MDSCs through CXCR2. Therapeutically, the combination of CXCR2 inhibitor SX-682 and anti-PD-1 treatment significantly extended survival in CRC mice and was more effective than SX-682 monotherapy. The results were replicated in head and neck cancer [38].

On the other hand, the researchers noted that differentially expressed IL23 and its downstream IL17 were associated with KRAS in a stage-specific fashion along CRC progression, accompanied by increased infiltration of MDSCs [39]. Yuan et al. explained that the IL23/IL17 axis may promote MDSCs infiltration through IL-1β-CXCL1 signaling. High levels of IL-1β were found in LUAD mouse models with KRAS G12D mutations, and IL-1β blocking significantly reduced the infiltration of PMN-MDSC in the lung. In vitro experiments and the TCGA database also confirmed the positive correlation between IL1B and CXCL1, a PMN-MDSC chemoattractant, at the transcriptional level [40].

Yet another example involves the initiation of pancreatic ductal adenocarcinoma (PDAC). KRAS-mutated pancreatic epithelial cells were susceptible to the peroxisome proliferator-activated receptor δ (PPARδ). Then M2 macrophages and MDSCs were attracted through the CCL2/CCR2 axis to accelerate the pancreatic intraepithelial neoplasia (PanIN) to PDAC [41].

### Cancer-associated fibroblasts

Cancer-associated fibroblasts (CAFs) are found in various proportions across the spectrum of carcinomas, constituting in many cases the preponderant cell population of the tumor stroma. Although CAFs do not belong to immune cells, their influence on immune cells cannot be ignored. On the one hand, CAFs inhibit immune cell function by secreting various cytokines and metabolites. On the other hand, CAFs can regulate the extracellular matrix and have a barrier effect on the infiltration of drugs and immune cells [42]. Accordingly, it is crucial to elucidate whether RAS signals are associated with tumor stromal response and CAFs.

**Fig. 2:**
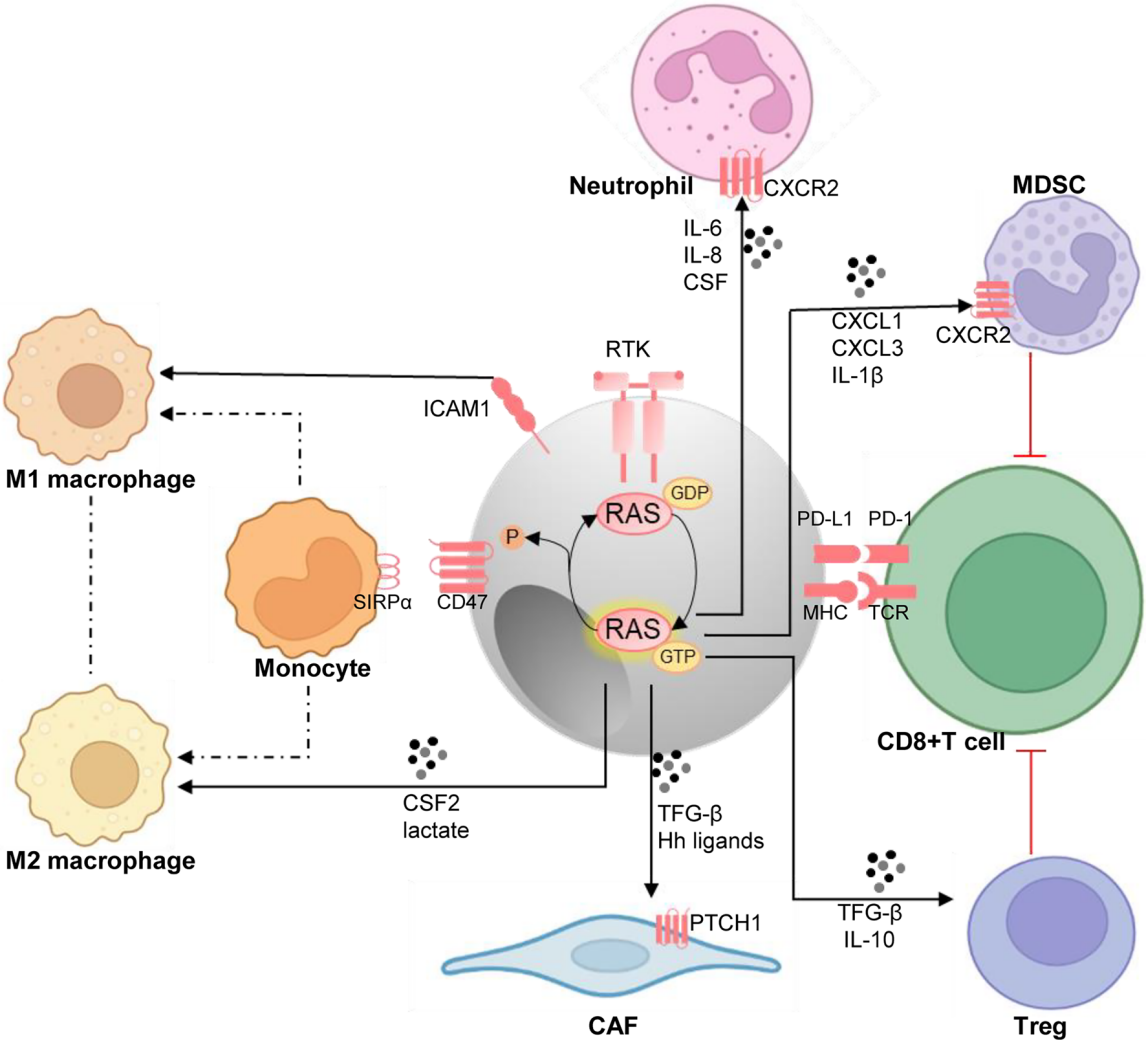
The influence of mutant RAS on the tumor immune microenvironment. RAS mutant cancer cells not only regulate tumor-associated immune response at the level of recruitment, activation, and differentiation of immune cells, but also induce cancer cells to escape immune surveillance via upregulating PD-L1 and CD47 signals. Together, these alterations shape an immunosuppressive state, and present opportunities for intervention in the treatment of RAS-mutated malignancies

As discussed above, KRAS G12D conduces to the pathogenesis of PDAC. Similarly, the gross stromal pancreatic stellate cell (PSC) expansion was observed in the KRAS G12D microenvironment. It is thought to act as a stromal response in the early stages of pancreatic cancer, shielding it from attack by the immune system. There is evidence that the association between RAS mutations and stromal responses is mediated by Hedgehog signaling. Only when RAS and Hedgehog are activated simultaneously can pancreatic tissue become cancerous [43–45]. Coincidentally, KRAS-mutated MSS CRC mouse models also showed strong stromal activation, with transforming growth factor-β (TGF-β) signaling thought to be dominant [46]. The above underlying mechanism is plausible because a large amount of evidence has confirmed that TGF- β and Hedgehog signals have overlapping effect activities and can regulate key components of each other’s pathways [47, 48] (Fig. 2).

## RAS mutation and immune escape

Tumor cells evolve a variety of strategies to limit or circumvent immune attack. In so doing, RAS mutation is undoubtedly an accomplice. It is increasingly evident that KRAS mutant cancer cells not only regulate tumor-associated immune response at the level of recruitment, activation, and differentiation of immune cells, but also induce cancer cells to escape immune surveillance. Amid this wealth of new knowledge, we highlight several advances of particular relevance to PD-L1 and CD47 (Fig. 2).

### PD-L1

The proposition that tumor cells evade immune surveillance by up-regulating PD-L1 expression has been widely recognized. Interferon-γ (IFN-γ) is an effective stimulus of PD-L1 expression. A typical example involved TANK-binding kinase 1 (TBK1), a phosphokinase essential for interferon regulatory factor activation. And TBK1 was believed to be regulated by the AXL-Ras-RalB signal [49]. Observations in PDAC hinted that TBK1 not only promoted malignant progression of tumors by driving epithelial mesenchymal transformation (EMT), but also assisted immune escape by inducing upregulation of PD-L1. This process was particularly obvious in the downstream of IFN-γ signal and involved autophagy and activation of JAK pathway. Treatment with TBK1 or JAK inhibitors could not only inhibit autophagy, but also reduce PD-L1 expression [50–52].

Similar to IFN-γ, 4-hydroxytamoxifen (4-OHT) induced oncogenic RAS signaling significantly increased PD-L1 expression on lung cancer cell after 48h. This phenomenon was not tissue-specific or RAS subtype-specific. And gentle stimulation over a long period of time was more effective. In this model, MEK inhibition reversed KRAS-mediated upregulation of PD-L1 mRNA, but PI3K inhibition only reduced PD-L1 protein expression, which confirmed the hypothesis of post-transcriptional regulation of PDL1 expression by MEK signal [53–55].

Human and mouse PD-L1 mRNA are known to be unstable transcripts. Mathieu et al. found a compelling case that RAS signaling increased the stability of PD-L1 mRNA by negatively regulating the AU-rich element-binding protein tristetraprolin (TTP). To put it simply, the mechanism involved was that carcinogenic RAS stimulated p38 signal transduction by promoting MEK-dependent ROS accumulation, thereby affecting TTP function [56, 57]. p38 signaling was known to be closely related to autophagy, and autophagy inhibitors or silencing ATG7 expression partially reversed the downregulation of PD-L1 caused by ERK inhibition [58, 59].

In addition, the association between KRAS and PD-L1 was also influenced by the synergistic promotion of other related gene mutations [60]. The highest level of PD-L1 was found in KRAS/TP53 co-mutated lung cancers, compared with wild-type and single-mutant types. Therefore, the KRAS/TP53 co-mutation can be used as a potential biomarker of ICB [61–63]. Mechanically, ARF6 and its effect-Amap1 were defined as the main targets of cooperative promotion of PD-L1 up-regulation in KRAS/ TP53-mutated pancreatic cancer [64].

Although a positive correlation between KRAS mutation and PD-L1 expression has been observed in lung and pancreatic cancer, PD-L1 expression has been associated with reduced frequency of KRAS mutation in CRC. Analysis within the TGCA-COAD database suggested that RAS mutations were enriched in patients with low expression of several inhibitory molecules, including PDL1, CTLA4, LAG3, and TIM3. Interestingly, the Albitar team tested the expression of PD-L1 in tissue samples of 107 CRC patients using next-generation sequencing (NGS). They found no correlation between PD-L1 expression and the mutant status of any RAS gene. Nor was there a correlation between TP53 mutation and PD-L1 expression [65]. This may be due to the existence of microsatellite instability in CRC, and a more exact mechanism remains a matter of debate [66].

### CD47

As two representative immune checkpoints that are often overexpressed on the surface of cancer cells, PD-L1 is responsible for evading attack by the adaptive immune system, while CD47 is the major contributor to resistance to innate immune phagocytosis by interacting with its ligands including platelet reactive protein-1 (TSP-1), signal-regulatory protein-α (SIRP-α), integrin, and protein tyrosine phosphatase substrate 1 (SHPS-1) carrying the SH2 domain [67, 68].

A recent study has shed some light: CD47 is regulated by KRAS signals. In LUAD patients and KRAS mutant lung cancer mice, the mutant KRAS activated PI3K/STAT3 signal transduction, thus inhibiting the post-transcriptional inhibition of CD47 by miR-34a. In treatment, destroying the KRAS/CD47 signal transduction axis with KRAS siRNA, KRAS G12C inhibitor or miR-34a mimics enhanced the phagocytosis of macrophages and restored innate immune surveillance.

Strategies to target CD47 have been developed in preclinical and clinical trials [69–71]. Given the widespread expression of CD47, anti-CD47 antibodies may cause serious side effects such as anemia, thrombocytopenia, and leukopenia, as observed in animal models [72, 73]. A significant attempt in the future is to combine KRAS inhibitors with anti-CD47 antibodies, which may help reduce side effects and toxicity.

## Treatment strategies for RAS-driven cancers

### Indirectly blocking of RAS signals

The RAS family has long been the most notorious of the undruggable targets. In 2021, Sotorasib (AMG-510) was approved for the treatment of patients with KRAS G12C mutation in NSCLC, becoming the first KRAS-targeted drug [74, 75]. Later, Adagrasib (MRTX849) came from behind to bring more superior clinical data in a variety of solid tumors [76].

Beforehand, blocking RAS oncogenic signals mainly focused on inhibiting membrane localization and upstream or downstream conduction. Previous studies have known that RAS proteins located on the cell membrane under the stability of PDEδ after being modified by farnesyltransferase (FTase) and/or geranylgeranyltransferase (GGTase). Although all RAS isoforms are substrates for FTase, HRAS are completely dependent upon farnesylation. Hence, inhibition of farnesylation or PDEδ seems to be an attractive therapeutic approach [10, 77, 78]. However, FTase has hundreds of substrates in human cells, including proteins involved in mitosis and the cytoskeleton [79]. Salirasib is a second-generation drug that inhibits the membrane localization of all activated RAS subtypes (KRAS, NRAS, and HRAS). A phase II trial of Salirasib in LUAD with KRAS mutations did not show any significant activity [80]. Further development of the drug has ceased.

In addition to inhibiting the membrane localization of RAS protein, the researchers also tried to block the upstream and downstream signal transduction of RAS. Since its discovery in 1992, SHP2 has emerged as a key upstream regulator of the RAS-MAPK signaling pathway. Typically, when SHP2 is activated by receptor tyrosine kinases (RTKs), it can recruit GRB2 and SOS1/2 to activate the RAS/MEK signal. A particularly large number of allosteric inhibitors have been identified with surprising inhibitory power against SHP2 [81]. SHP099 is the first effective, selective and orally bioavailable allosteric SHP2 inhibitor and has demonstrated strong antitumor activity in mouse xenograft models without significant toxicity or side effects [82].

SOS1 and its byproduct SOS2 are very important members of RAS-GEFs. A representative small molecule inhibitor is BI-3406, which can selectively bind to the active site of SOS1 and reduce the formation of GTP-RAS [83]. In addition, based on the theory that the negative feedback of SOS1 modulates RAS signaling biphasically, several novel agents were developed for preclinical models, such as agonist-Based SOS1 PROTACs [84] and SOS1 instigate-based degraders P7 [85], and showed an encouraging antitumor effect.

**Fig. 3:**
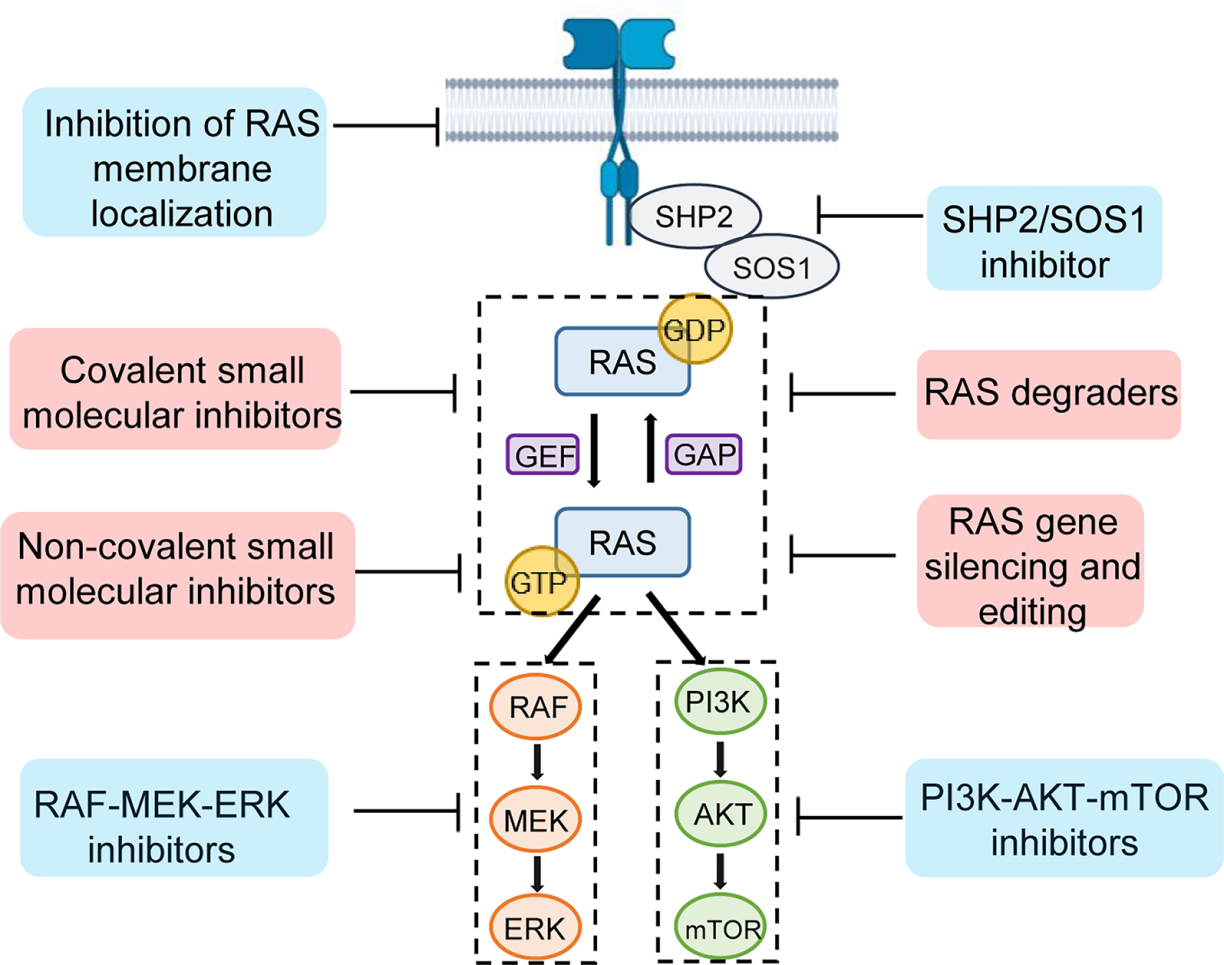
RAS signaling pathways with targeting strategies. RAS mutations mainly lead to uncontrolled activation of the MAP kinase pathway and PI3K pathway. Indirect strategies for targeting RAS include the inhibition of RAS membrane localization, suppression of SHP2/SOS1 and blockage of downstream signal. Direct strategies for targeting RAS include small molecular inhibitors for RAS proteins, genetic engineering technology and RAS degraders

Cumulative evidence points to RAS-regulated RAF-MEK-ERK pathways and PI3Kα-AKT-mTOR pathways as major drivers of tumorigenicity. Selumetinib (AZD6244) is an oral selective allosteric inhibitor of MEK1 and MEK2 kinase [86]. AZD0364 is a potent and selective inhibitor of ERK1 and ERK2. Flemington et al. expounded that AZD0364 combined with selumetinib (AZD6244) produced deeper and longer-lasting tumor suppressive effects in a xenograft model [87]. Alpelisib (BYL719) is a selective inhibitor of PI3Kα. A phase Ia study (NCT01219699) defined the safety of single-agent alpelisib (BYL719) in solid tumors, and subsequent clinical efficacy is still expected [88]. Similarly, the combination of Selumetinib and BYL719 showed a synergistic effect in inhibiting the growth of A549 xenograft tumors [89] (Fig. 3, Table 1).

**Table 1:** RAS targeted therapy strategies for cancer

Classification	Strategies	Mechanism	Examples	References
Indirectly blocking of RAS signals	Inhibition of RAS membrane localization	Inhibition of farnesylation or PDEδ	Lonafarnib Tipifarnib Salirasib	[10, 83, 84, 86]
	Inhibition of RAS signaling pathway transduction	SHP2 inhibitors	SHP099 TNO155 RMC-4630	[87–89]
		SOS1 inhibitors	BAY-293 BI-3406	[90–92]
		RAF-MEK-ERK inhibitors	LXH-254 Selumetinib (AZD6244) Tizaterkib (AZD0364)	[93, 94]
		PI3K-AKT-mTOR inhibitors	Alpelisib (BYL719) Lpatasertib Everolimus	[95, 96]
Direct targeting of RAS proteins	Small molecular inhibitors (covalent interactions)	cysteine pocket for KRAS G12C	Sotorasib Adagrasib	[80–82]
		arginase pocket for KRAS G12R	α,β-Diketoamide ligand	[97]
		serine pocket for KRAS G12S	β-Lactone ligand	[98]
	Small molecular inhibitors (non-covalent interactions)	Triple complex with KRAS G12D and CypA	RMC9805	[99]
		Salt bridge for KRAS G12D	MRTX-1133 TH-Z835	[100–102]
	RAS gene silencing and editing	RNAi CRISPR	siRNAs, shRNAs, CRISPR/Cas9 (DNA), CRISPR/Cas13 (RNA)	[105–108]
	RAS degraders	PROTAC, linker-based degraders, direct proteolysis degraders	LC-2 KP-14	[109–112]

### Direct targeting of RAS proteins

In the last decade, concerted efforts in therapeutic modeling, drug exploitation, and key finding of cysteine-binding pocket in KRAS G12C protein have ushered in a new era of tumor therapy targeting RAS. In addition to Sotorasib and Adagrasib mentioned above, several other KRAS G12C inhibitors with similar mechanisms have entered clinical development, such as GDC-6036 (NCT04449874), JDQ443 (NCT04699188), and D-1553 (NCT04585035).

Notably, the need to target other KRAS mutations is greater, with a higher frequency than G12C. The chemical development of other covalent ligands of mutant arginine residue in KRAS G12R and mutant serine residue in KRAS G12S provided a novel therapeutic window specifically for KRAS-driven cancers [90, 91], although in vivo experimental evidence was incomplete. John et al. designed a new three-complex structure: KRAS-CypA-RMC-9805, which blocked GTP-bound KRAS G12D with lower off-target performance [92]. MRTX-1133 is a non-covalent KRAS G12D inhibitor, which is effective in animal models, but its clinical data have not been published [93]. A notable recent breakthrough was the discovery of the pharmacological mechanism of TH-Z835. It acts as an inhibitor by forming a salt bridge between its own piperazine portion and the Asp12 residue of the KRASG12D mutant protein. And even more surprising, the mouse xenotransplantation model of pancreatic cancer showed that TH-Z835 significantly reduced tumor volume and synergistically acted with anti-PD-1 antibody [94]. Among the broad-spectrum KRAS inhibitors, BI 2852 binds to KRAS between switch I and II with nanomolar affinity. In doing so, it blocked all GEFs, GAPs, and effectors, completely silencing the KRAS signal [95, 96]. RMC-6236 is a broad-spectrum inhibitor targeting all mutants as well as wild-type KRAS, HRAS, and NRAS. A Phase I clinical trial of RMC-6236 is currently underway (NCT5379985).

In addition to the above small molecule inhibitors, mutant RAS gene silencing and editing also seem to be theoretically plausible. Mutant RAS-specific siRNAs or shRNAs and CRISPR/Cas9 (DNA) or Cas13 (RNA) systems are attempting to make waves in optimizing treatments for RAS-driven cancers [97–100], even in the absence of sufficient clinical data on safety and feasibility (NCT01188785, NCT01676259, NCT03608631). More recently, advanced efforts in RAS degraders including proteolysis-targeting chimera (PROTAC), linker-based degraders, and direct proteolysis degraders have been explored as new strategies [101, 102]. LC-2 has been reported to be the first PROTAC capable of inducing endogenous KRAS G12C degradation by recruiting VHLs [103]. Compound KP-14, a degrader synthesized based on KRAS G12C-IN-3 and pomalidomide, significantly weakened the proliferation ability of lung cancer cells NCI-H358 in vitro [104] (Fig. 3, Table 1).

Although the practice of targeting RAS mutations is a great breakthrough, the dilemma that clinical oncologists still face is the emergence of drug resistance, including innate and acquired resistance [105, 106]. Therefore, the exploration of optimal combination strategies and the development of novel therapies with advanced technologies are hot spots in the futher.

## Immunotherapy regimens targeting RAS

### Cancer vaccines

#### Peptide-based vaccines

The mutant RAS proteins are cancer-specific neoepitopes that are recognized by autologous T cells and constitute ideal cancer vaccine targets. Clinical trials of KRAS peptide vaccines can be traced back to the 1990s [107]. These initial trials have highlighted the safety of the peptide vaccines, but have shown only slight clinical benefits. It can be said that a single peptide-based vaccine cannot overcome the immunosuppression caused by mutated RAS.

Several attempts have made progress in recent years, including combining RAS vaccines with other methods or modifying them. Gemcitabine is the standard treatment for patients with advanced pancreatic cancer. TG01 is a peptide vaccine for the treatment of patients with solid mutated RAS cancer. A phase I/II trial involving 32 patients with pancreatic cancer suggested that gemcitabine combined with TG-01 had a positive survival benefit in postoperative adjuvant therapy for RAS-mutated pancreatic cancer patients [108]. Comutations are common in tumors, and the peptide vaccination targeting multiple mutated sites therefore may elicit a polyvalent, multifunctional, and curative effect. Jasmin et al. engineered a panel of long peptides (28–35 aa) containing TP35 R248W and KRAS G12V mutations. It was found that the vaccine containing both mutated proteins induced a significantly higher T cell response than the corresponding wild sequences [109].

Other evidence has accumulated pointing to the interference of lipid metabolism with RAS peptide vaccines. Avasimibe is a cholesterol acyltransferase inhibitor [110]. Previous studies have prompted that avasimibe significantly increases intracellular free cholesterol levels, thereby leading to apoptosis and inhibiting proliferation in a variety of human tumor cell lines [111–113]. Yet another example involved avasimibe affecting the function of CD8 T cells. The researchers found that avasimibe-mediated elevated cholesterol levels increased immune synapse formation and T cell receptor signal transduction in CD8 T cells, which in turn enhanced CD8T cell-dependent anti-tumor response [114]. Considering this, Pan et al. designed to use the RAS vaccine in combination with avasimibe, which significantly increased the abundance of CD8+ T cell infiltration and the levels of IFN-γ and granzyme B in lung cancer mouse models, playing a synergistic role [115]. Additionally, Arbelaez et al. designed a novel cationic lipoplexes delivery system to test the efficacy of a long peptide vaccine carrying KRAS G12D. The observation was surprising [116].

### DC-based vaccines

Based on the strong antigen presentation activity and T cell activation characteristics, DC vaccine technology is also gradually mature [117]. An illuminating example involved GI-4000. It was found that DC could absorb the mutant KRAS peptide produced by recombinant yeast and undergo maturation. GI-4000 is a heat-inactivated, engineered DC-based vaccine that is genetically engineered to express RAS G12 (G12V, G12C, G12D, or G12R) and Q61 (Q61L and Q61R) mutations [118]. Multiple lines of evidence indicated that GI-4000 demonstrated excellent safety and immunogenicity in most subjects with lung, colorectal, or pancreatic cancers [119]. In addition, progress has been made in preclinical trials of DC vaccine in combination with conventional chemotherapy drugs. Kondui et al. developed a DC vaccine, loaded with mRNA and lysate derived from Kras^G12D^p53^−/−^luc2^neg^ cells. The combination of gemcitabine and DC vaccine promoted in situ tumor eradication in Krasg12D-mutated PDAC mice and prevented metastasis and recurrence [120].

#### DNA and mRNA vaccines

**Fig. 4:**
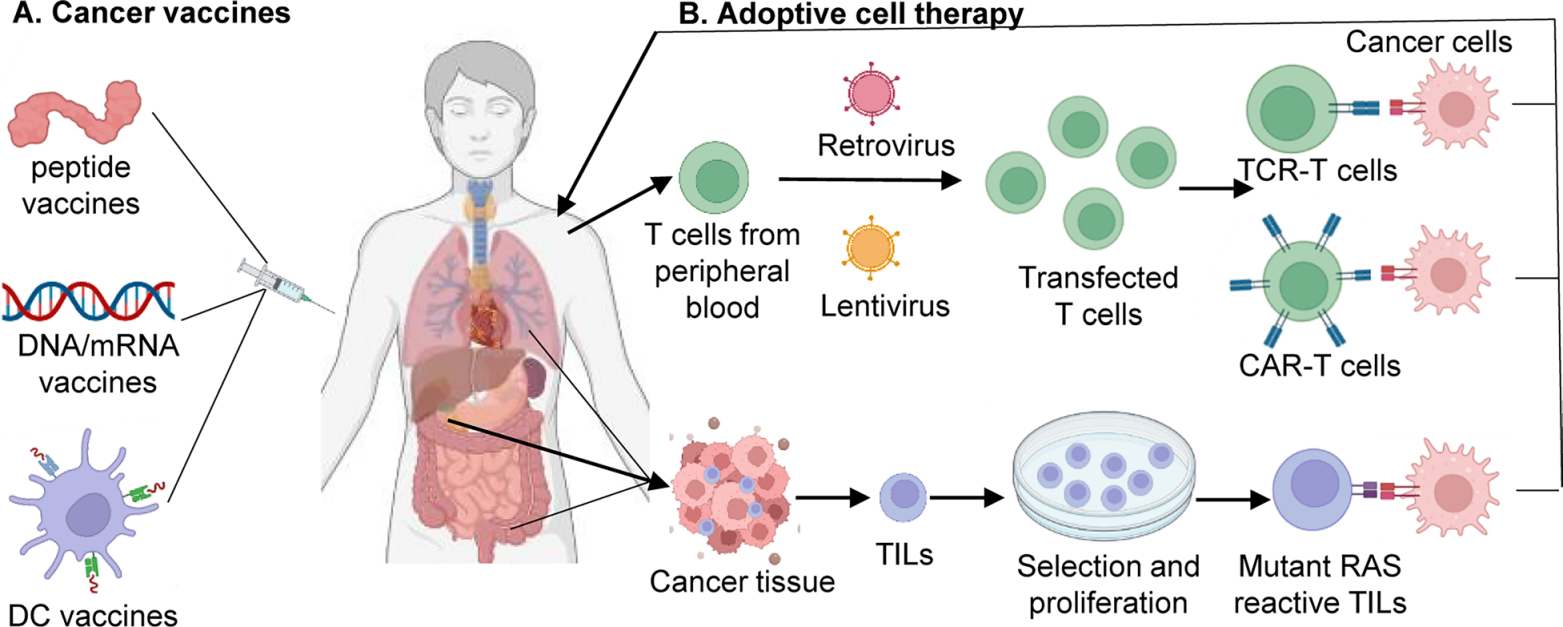
Immunotherapy regimens for RAS mutant cancers. **A** By injecting peptide, mRNA, and DC-based vaccines, the activated T cell receptor (TCR) binds to the major histocompatibility complex (MHC). The powerful antigen–antibody response eventually leads to cancer cell death. **B** Peripheral blood T cells from cancer patients are genetically modified by viral vectors to create CAR-T and TCR-T cells that express patient-specific, RAS-driven cancer cell neoantigens. The patient-specific tumor-infiltration active T cells (TILs) are isolated and expanded ex vivo. The most tumor-specific and functionally enriched T cells are administered to the patient once lymphodepletion

DNA vaccines are another major breakthrough in genetic engineering technology. DNA vaccines usually use plasmids, viruses, or bacteria as vectors to enhance stability and delivery efficiency. The past decade has witnessed an astonishing outpouring of research on DNA vaccines [121]. However, mRNA vaccines have attracted the most attention due to high potency, safe administration, rapid development potential, and cost-effective manufacturing [122, 123]. In 2018, Modena and Merck teamed up to create a new shared antigen vaccine therapy called mRNA-5671, where mRNA was designed to encode KRAS mutations (G12D, G12V, G13D, and G12C). In preclinical trials, MRNA-5671 enhanced the response of CD8T cells to mRNA encoding mutated KRAS [124]. Phase I trials of mRNA-5671 are currently underway in two groups, either as monotherapy or in combination with the PD-1 inhibitor pembrolizumab (NCT03948763). We are eagerly awaiting the results (Fig. 4).

### Adoptive cell therapy

Combined with the above, the well-known challenges of targeting RAS-mutated tumors are inadequate or dysfunctional immune cell infiltration and avoidance of immune surveillance. Adoptive cell transfer, the infusion of large numbers of activated autologous lymphocytes, alleviates these concerns. CD8+ T cells transfused with targeted mutant KRAS showed effective antitumor effects in KRAS G12D metastatic colorectal cancer, but were limited by HLA-C * 08:02 [125]. In addition, TCR9a to 9c recognized G12D nonamer with multiple conserved contacts through shared CDR2β and CDR3α, generating a high-affinity oligoclonal response [126]. This study provided an innovative structural approach to support adoptive therapy.

More recently, advances in genetic engineering have led to the evolution of more precise and efficient chimeric antigen receptor T cell (CAR-T) therapies. This therapy bypasses the MHC/TCR-mediated T cell response in cancer cells and does not face any intra tumor heterogeneity [127, 128]. The clinical application of CAR T cell therapy in hematologic malignancies is very promising [129, 130]. Ever more powerful experimental and computational technologies are providing an avalanche of “big data” about neoantigens, which are used in the design of CAR-T therapies [131]. An admittedly incomplete representation of carcinoembryonic antigen (CEA) and mesothelin is highlighted below (Fig. 3).

CEA is a valuable target for adoptive cell therapy and is upregulated in RAS mutation-induced tumors, such as colorectal and pancreatic cancers. Anti-CEA CAR-T cell therapy in CRC patients, although mediating regression of metastatic colorectal cancer, induced severe transient inflammatory colitis due to CEA expression in healthy intestinal epithelium [132]. Nevertheless, substantial autoimmunity was not present in the CEA transgenic (CEA-tg) mice, which perfectly mimics the human condition with respect to physiological expression, solubility in serum, and immune tolerance of CEA. Markus et al. verified that the injection of the anti-CEA CAR-T cells specifically and efficiently inhibited pancreas carcinoma and produced long-term tumor eradication in 67% of CEAtg mice [133]. Although promising in preclinical studies, CEA targeting CAR-T cell therapy has not provided the expected success in phase I dose-escalation trials. In addition, the second dose caused pulmonary toxicity. Thus, the test was terminated (CRUKD/07/064).

Mesothelin is a cell surface molecule that is upregulated in over 80% of epithelial cancers, including PDAC and lung adenocarcinoma [134]. Mesothelin-redirected CAR-T cell (CAR-T meso) therapy has shown some efficacy in animal models, but has little clinical benefit [135]. A phase I study (NCT03054298) of meso-CAR T therapy in advanced solid cancers is still ongoing. Keisuke et al. combined meso-CAR T cells with oncolytic adenoviruses expressing TNF-α and IL-2, which not only controlled the progression of in situ tumor in PDA mice driven by KRAS mutation, but also inhibited tumor metastasis, showing a strong clinical prospect [136].

## Conclusions

This review concluded the direct and indirect treatment strategies for RAS mutations. In addation, we discussed advances in RAS**-**targeted immunotherapies, including cancer vaccines and adoptive cell therapies**.** While a detailed discussion on new treatments such as RAS gene silencing and editing, as well as protein degraders is beyond the scope of this review, this space will need to be watched closely in the future. Over time, treatment strategies with personalized therapy and great potential to achieve lasting clinical benefits are hopeful.

## Data Availability

Not applicable.

## Abbreviations

CAF: Cancer-associated fibroblast
CAR-T: Chimeric antigen receptor T cell
CEA: Carcinoembryonic antigen
CRC: Colorectal cancer
EMT: Epithelial mesenchymal transformation
GAP: GTPase activating protein
GEF: Guanine exchange factor
ICAM-1: Intercellular adhesion molecule-1
IFN-γ: Interferon-γ
IRF: Interferon regulatory factor
MDSC: Myeloid-derived suppressor cell
NET: Neutrophil extracellular trap
NSCLC: Non small cell lung cancer
ORR: Objective response rate
PDAC: Pancreatic ductal adenocarcinoma
PPARδ: Peroxisome proliferator-activated receptor δ
PROTAC: Proteolysis-targeting chimera
RAS: Rat sarcoma virus
RTK: Receptor tyrosine kinase
SHP2: Srchomology-2-domain-containing PTP 2
SOS1: Son of sevenless 1
TAM: Tumor-associated macrophage
TBK1: TANK-binding kinase 1
TGF-β: Transforming growth factor-β
TIM: Tumor immune microenvironment
Treg: Regulatory T cell

## References

[1] Hanahan D, Weinberg RA (2011). Hallmarks of cancer: the next generation. Cell.

[2] Sealover NE, Kortum RL (2022). Heterogeneity in RAS mutations: one size does not fit all. Sci Signal.

[3] Dias Carvalho P, Guimarães CF, Cardoso AP, Mendonça S, Costa ÂM, Oliveira MJ (2018). KRAS oncogenic signaling extends beyond cancer cells to orchestrate the microenvironment. Cancer Res.

[4] Aleksakhina SN, Imyanitov EN (2021). Cancer therapy guided by mutation tests: current status and perspectives. Int J Mol Sci.

[5] Nissley DV, McCormick F (2022). RAS at 40: update from the RAS initiative. Cancer Discov.

[6] Hidalgo F, Nocka LM, Shah NH, Gorday K, Latorraca NR, Bandaru P (2022). A saturation-mutagenesis analysis of the interplay between stability and activation in Ras. Elife.

[7] Burge RA, Hobbs GA (2022). Not all RAS mutations are equal: a detailed review of the functional diversity of RAS hot spot mutations. Adv Cancer Res.

[8] Li S, Balmain A, Counter CM (2018). A model for RAS mutation patterns in cancers: finding the sweet spot. Nat Rev Cancer.

[9] Randic T, Kozar I, Margue C, Utikal J, Kreis S (2021). NRAS mutant melanoma: towards better therapies. Cancer Treat Rev.

[10] Ho AL, Brana I, Haddad R, Bauman J, Bible K, Oosting S (2021). Tipifarnib in head and neck squamous cell carcinoma with HRAS mutations. J Clin Oncol.

[11] Jung J, Cho K-J, Naji AK, Clemons KN, Wong CO, Villanueva M (2019). HRAS-driven cancer cells are vulnerable to TRPML1 inhibition. EMBO Rep.

[12] Khan AQ, Kuttikrishnan S, Siveen KS, Prabhu KS, Shanmugakonar M, Al-Naemi HA (2019). RAS-mediated oncogenic signaling pathways in human malignancies. Seminars in cancer biology.

[13] Dubey S, Ghosh S, Goswami D, Ghatak D, De R (2023). Immunometabolic attributes and mitochondria-associated signaling of tumor-associated macrophages in tumor microenvironment modulate cancer progression. Biochem Pharmacol.

[14] Mantovani A, Allavena P, Marchesi F, Garlanda C (2022). Macrophages as tools and targets in cancer therapy. Nat Rev Drug Discov.

[15] Liou G-Y, Döppler H, Necela B, Edenfield B, Zhang L, Dawson DW (2015). Mutant KRAS-induced expression of ICAM-1 in pancreatic acinar cells causes attraction of macrophages to expedite the formation of precancerous lesions. Cancer Discov.

[16] Storz P (2015). The crosstalk between acinar cells with Kras mutations and M1-polarized macrophages leads to initiation of pancreatic precancerous lesions. Oncoimmunology.

[17] Dai E, Han L, Liu J, Xie Y, Kroemer G, Klionsky DJ (2020). Autophagy-dependent ferroptosis drives tumor-associated macrophage polarization via release and uptake of oncogenic KRAS protein. Autophagy.

[18] Liu H, Liang Z, Zhou C, Zeng Z, Wang F, Hu T (2021). Mutant KRAS triggers functional reprogramming of tumor-associated macrophages in colorectal cancer. Signal Transduct Target Ther.

[19] Quail DF, Amulic B, Aziz M, Barnes BJ, Eruslanov E, Fridlender ZG (2022). Neutrophil phenotypes and functions in cancer: a consensus statement. J Exp Med.

[20] Sunaga N, Imai H, Shimizu K, Shames DS, Kakegawa S, Girard L (2012). Oncogenic KRAS-induced interleukin-8 overexpression promotes cell growth and migration and contributes to aggressive phenotypes of non-small cell lung cancer. Int J Cancer.

[21] de Andrea CE, Ochoa MC, Villalba-Esparza M, Teijeira Á, Schalper KA, Abengozar-Muela M (2021). Heterogenous presence of neutrophil extracellular traps in human solid tumours is partially dependent on IL-8. J Pathol.

[22] Teijeira A, Garasa S, Ochoa MC, Villalba M, Olivera I, Cirella A (2021). IL8, neutrophils, and NETs in a collusion against cancer immunity and immunotherapy. Clin Cancer Res.

[23] Shang A, Gu C, Zhou C, Yang Y, Chen C, Zeng B (2020). Exosomal KRAS mutation promotes the formation of tumor-associated neutrophil extracellular traps and causes deterioration of colorectal cancer by inducing IL-8 expression. Cell Commun Signal.

[24] Akbay EA, Koyama S, Liu Y, Dries R, Bufe LE, Silkes M (2017). Interleukin-17A promotes lung tumor progression through neutrophil attraction to tumor sites and mediating resistance to PD-1 blockade. J Thorac Oncol.

[25] Mills KHG (2023). IL-17 and IL-17-producing cells in protection versus pathology. Nat Rev Immunol.

[26] Ritzmann F, Lunding LP, Bals R, Wegmann M, Beisswenger C (2022). IL-17 cytokines and chronic lung diseases. Cells.

[27] Kitajima S, Tani T, Springer BF, Campisi M, Osaki T, Haratani K (2022). MPS1 inhibition primes immunogenicity of KRAS-LKB1 mutant lung cancer. Cancer Cell.

[28] Kitajima S, Ivanova E, Guo S, Yoshida R, Campisi M, Sundararaman SK (2019). Suppression of STING associated with LKB1 loss in KRAS-driven lung cancer. Cancer Discov.

[29] Koyama S, Akbay EA, Li YY, Aref AR, Skoulidis F, Herter-Sprie GS (2016). STK11/LKB1 deficiency promotes neutrophil recruitment and proinflammatory cytokine production to suppress T-cell activity in the lung tumor microenvironment. Cancer Res.

[30] Wing JB, Tanaka A, Sakaguchi S (2019). Human FOXP3+ regulatory T cell heterogeneity and function in autoimmunity and cancer. Immunity.

[31] Zdanov S, Mandapathil M, Abu Eid R, Adamson-Fadeyi S, Wilson W, Qian J (2016). Mutant KRAS conversion of conventional T cells into regulatory T cells. Cancer Immunol Res.

[32] Domvri K, Petanidis S, Zarogoulidis P, Anestakis D, Tsavlis D, Bai C (2021). Treg-dependent immunosuppression triggers effector T cell dysfunction via the STING/ILC2 axis. Clin Immunol.

[33] Wu L, Zhao W, Tang S, Chen R, Ji M, Yang X (2022). Role of ILC2s in solid tumors: facilitate or inhibit?. Front Immunol.

[34] Liu J, Huang X, Liu H, Wei C, Ru H, Qin H (2021). Immune landscape and prognostic immune-related genes in KRAS-mutant colorectal cancer patients. J Transl Med.

[35] Hossain DMS, Panda AK, Chakrabarty S, Bhattacharjee P, Kajal K, Mohanty S (2015). MEK inhibition prevents tumour-shed transforming growth factor-β-induced T-regulatory cell augmentation in tumour milieu. Immunology.

[36] Hegde S, Leader AM, Merad M (2021). MDSC: markers, development, states, and unaddressed complexity. Immunity.

[37] Liao W, Overman MJ, Boutin AT, Shang X, Zhao D, Dey P (2019). KRAS-IRF2 axis drives immune suppression and immune therapy resistance in colorectal cancer. Cancer Cell.

[38] Greene S, Robbins Y, Mydlarz WK, Huynh AP, Schmitt NC, Friedman J (2020). Inhibition of MDSC trafficking with SX-682, a CXCR1/2 inhibitor, enhances NK-cell immunotherapy in head and neck cancer models. Clin Cancer Res.

[39] Petanidis S, Anestakis D, Argyraki M, Hadzopoulou-Cladaras M, Salifoglou A (2013). Differential expression of IL-17, 22 and 23 in the progression of colorectal cancer in patients with K-ras mutation: Ras signal inhibition and crosstalk with GM-CSF and IFN-γ. PLoS ONE.

[40] Yuan B, Clowers MJ, Velasco WV, Peng S, Peng Q, Shi Y (2022). Targeting IL-1β as an immunopreventive and therapeutic modality for K-ras-mutant lung cancer. JCI Insight.

[41] Liu Y, Deguchi Y, Wei D, Liu F, Moussalli MJ, Deguchi E (2022). Rapid acceleration of KRAS-mutant pancreatic carcinogenesis via remodeling of tumor immune microenvironment by PPARδ. Nat Commun.

[42] Luo H, Xia X, Huang L-B, An H, Cao M, Kim GD (2022). Pan-cancer single-cell analysis reveals the heterogeneity and plasticity of cancer-associated fibroblasts in the tumor microenvironment. Nat Commun.

[43] Hingorani SR (2023). Epithelial and stromal co-evolution and complicity in pancreatic cancer. Nat Rev Cancer.

[44] Shinkawa T, Ohuchida K, Nakamura M (2022). Heterogeneity of cancer-associated fibroblasts and the tumor immune microenvironment in pancreatic cancer. Cancers.

[45] Bryce AS, Dreyer SB, Froeling FEM, Chang DK (2022). Exploring the biology of cancer-associated fibroblasts in pancreatic cancer. Cancers.

[46] Tauriello DVF, Palomo-Ponce S, Stork D, Berenguer-Llergo A, Badia-Ramentol J, Iglesias M (2018). TGFβ drives immune evasion in genetically reconstituted colon cancer metastasis. Nature.

[47] Perrot CY, Javelaud D, Mauviel A (2013). Overlapping activities of TGF-β and hedgehog signaling in cancer: therapeutic targets for cancer treatment. Pharmacol Ther.

[48] Pelullo M, Zema S, Nardozza F, Checquolo S, Screpanti I, Bellavia D (2019). Wnt, Notch, and TGF-β pathways impinge on hedgehog signaling complexity: an open window on cancer. Front Genet.

[49] Cruz VH, Arner EN, Du W, Bremauntz AE, Brekken RA (2019). Axl-mediated activation of TBK1 drives epithelial plasticity in pancreatic cancer. JCI Insight.

[50] Yang S, Imamura Y, Jenkins RW, Cañadas I, Kitajima S, Aref A (2016). Autophagy inhibition dysregulates TBK1 signaling and promotes pancreatic inflammation. Cancer Immunol Res.

[51] Zhu L, Li Y, Xie X, Zhou X, Gu M, Jie Z (2019). TBKBP1 and TBK1 form a growth factor signalling axis mediating immunosuppression and tumourigenesis. Nat Cell Biol.

[52] Zhu Z, Aref AR, Cohoon TJ, Barbie TU, Imamura Y, Yang S (2014). Inhibition of KRAS-driven tumorigenicity by interruption of an autocrine cytokine circuit. Cancer Discov.

[53] Alspach E, Lussier DM, Schreiber RD (2019). Interferon γ and its important roles in promoting and inhibiting spontaneous and therapeutic cancer immunity. Cold Spring Harb Perspect Biol.

[54] Zhao R, Song Y, Wang Y, Huang Y, Li Z, Cui Y (2019). PD-1/PD-L1 blockade rescue exhausted CD8+ T cells in gastrointestinal stromal tumours via the PI3K/Akt/mTOR signalling pathway. Cell Prolif.

[55] Falcomatà C, Bärthel S, Widholz SA, Schneeweis C, Montero JJ, Toska A (2022). Selective multi-kinase inhibition sensitizes mesenchymal pancreatic cancer to immune checkpoint blockade by remodeling the tumor microenvironment. Nat Cancer.

[56] Coelho MA, de CarnéTrécesson S, Rana S, Zecchin D, Moore C, Molina-Arcas M (2017). Oncogenic RAS signaling promotes tumor immunoresistance by stabilizing PD-L1 mRNA. Immunity.

[57] Wu M-F, Huang Y-H, Chiu L-Y, Cherng S-H, Sheu G-T, Yang T-Y (2022). Curcumin induces apoptosis of chemoresistant lung cancer cells via ROS-regulated p38 MAPK phosphorylation. Int J Mol Sci.

[58] Sui X, Kong N, Ye L, Han W, Zhou J, Zhang Q (2014). p38 and JNK MAPK pathways control the balance of apoptosis and autophagy in response to chemotherapeutic agents. Cancer Lett.

[59] Gao Z, Chen J-F, Li X-G, Shi Y-H, Tang Z, Liu W-R (2022). KRAS acting through ERK signaling stabilizes PD-L1 via inhibiting autophagy pathway in intrahepatic cholangiocarcinoma. Cancer Cell Int.

[60] Skoulidis F, Byers LA, Diao L, Papadimitrakopoulou VA, Tong P, Izzo J (2015). Co-occurring genomic alterations define major subsets of KRAS-mutant lung adenocarcinoma with distinct biology, immune profiles, and therapeutic vulnerabilities. Cancer Discov.

[61] Dong Z-Y, Zhong W-Z, Zhang X-C, Su J, Xie Z, Liu S-Y (2017). Potential predictive value of TP53 and KRAS mutation status for response to PD-1 blockade immunotherapy in lung adenocarcinoma. Clin Cancer Res.

[62] Assoun S, Theou-Anton N, Nguenang M, Cazes A, Danel C, Abbar B (2019). Association of TP53 mutations with response and longer survival under immune checkpoint inhibitors in advanced non-small-cell lung cancer. Lung Cancer.

[63] Fang C, Zhang C, Zhao W-Q, Hu W-W, Wu J, Ji M (2019). Co-mutations of TP53 and KRAS serve as potential biomarkers for immune checkpoint blockade in squamous-cell non-small cell lung cancer: a case report. BMC Med Genom.

[64] Hashimoto S, Furukawa S, Hashimoto A, Tsutaho A, Fukao A, Sakamura Y (2019). ARF6 and AMAP1 are major targets of KRAS and TP53 mutations to promote invasion, PD-L1 dynamics, and immune evasion of pancreatic cancer. Proc Natl Acad Sci USA.

[65] Lal N, Beggs AD, Willcox BE, Middleton GW (2015). An immunogenomic stratification of colorectal cancer: Implications for development of targeted immunotherapy. Oncoimmunology.

[66] Marginean EC, Melosky B (2018). Is there a role for programmed death ligand-1 testing and immunotherapy in colorectal cancer with microsatellite instability? Part II-the challenge of programmed death ligand-1 testing and its role in microsatellite instability-high colorectal cancer. Arch Pathol Lab Med.

[67] Jiang Z, Sun H, Yu J, Tian W, Song Y (2021). Targeting CD47 for cancer immunotherapy. J Hematol Oncol.

[68] Jia X, Yan B, Tian X, Liu Q, Jin J, Shi J (2021). CD47/SIRPα pathway mediates cancer immune escape and immunotherapy. Int J Biol Sci.

[69] Sikic BI, Lakhani N, Patnaik A, Shah SA, Chandana SR, Rasco D (2019). First-in-human, first-in-class phase I trial of the anti-CD47 antibody Hu5F9-G4 in patients with advanced cancers. J Clin Oncol.

[70] Theruvath J, Menard M, Smith BAH, Linde MH, Coles GL, Dalton GN (2022). Anti-GD2 synergizes with CD47 blockade to mediate tumor eradication. Nat Med.

[71] Upton R, Banuelos A, Feng D, Biswas T, Kao K, McKenna K (2021). Combining CD47 blockade with trastuzumab eliminates HER2-positive breast cancer cells and overcomes trastuzumab tolerance. Proc Natl Acad Sci USA.

[72] Gwag T, Ma E, Zhou C, Wang S (2022). Anti-CD47 antibody treatment attenuates liver inflammation and fibrosis in experimental non-alcoholic steatohepatitis models. Liver Int.

[73] Ni H, Cao L, Wu Z, Wang L, Zhou S, Guo X (2022). Combined strategies for effective cancer immunotherapy with a novel anti-CD47 monoclonal antibody. Cancer Immunol Immunother.

[74] Hong DS, Fakih MG, Strickler JH, Desai J, Durm GA, Shapiro GI (2020). KRASG12C inhibition with sotorasib in advanced solid tumors. N Engl J Med.

[75] Skoulidis F, Li BT, Dy GK, Price TJ, Falchook GS, Wolf J (2021). Sotorasib for lung cancers with KRAS p.G12C mutation. N Engl J Med.

[76] Jänne PA, Riely GJ, Gadgeel SM, Heist RS, Ou S-HI, Pacheco JM (2022). Adagrasib in non-small-cell lung cancer harboring a KRASG12C mutation. N Engl J Med.

[77] Casique-Aguirre D, Briseño-Díaz P, García-Gutiérrez P, la Rosa CHG-D, Quintero-Barceinas RS, Rojo-Domínguez A (2018). KRas4B-PDE6δ complex stabilization by small molecules obtained by virtual screening affects Ras signaling in pancreatic cancer. BMC Cancer.

[78] Marchwicka A, Kamińska D, Monirialamdari M, Błażewska KM, Gendaszewska-Darmach E (2022). Protein prenyltransferases and their inhibitors: structural and functional characterization. Int J Mol Sci.

[79] Porcu G, Wilson C, Di Giandomenico D, Ragnini-Wilson A (2010). A yeast-based genomic strategy highlights the cell protein networks altered by FTase inhibitor peptidomimetics. Mol Cancer.

[80] Riely GJ, Johnson ML, Medina C, Rizvi NA, Miller VA, Kris MG (2011). A phase II trial of Salirasib in patients with lung adenocarcinomas with KRAS mutations. J Thorac Oncol.

[81] Xie J, Si X, Gu S, Wang M, Shen J, Li H (2017). Allosteric inhibitors of SHP2 with therapeutic potential for cancer treatment. J Med Chem.

[82] Chen Y-NP, LaMarche MJ, Chan HM, Fekkes P, Garcia-Fortanet J, Acker MG (2016). Allosteric inhibition of SHP2 phosphatase inhibits cancers driven by receptor tyrosine kinases. Nature.

[83] Hofmann MH, Gmachl M, Ramharter J, Savarese F, Gerlach D, Marszalek JR (2021). BI-3406, a potent and selective SOS1-KRAS interaction inhibitor, is effective in KRAS-driven cancers through combined MEK inhibition. Cancer Discov.

[84] Zhou C, Fan Z, Zhou Z, Li Y, Cui R, Liu C (2022). Discovery of the first-in-class agonist-based SOS1 PROTACs effective in human cancer cells harboring various KRAS mutations. J Med Chem.

[85] Bian Y, Alem D, Beato F, Hogenson TL, Yang X, Jiang K (2022). Development of SOS1 inhibitor-based degraders to target KRAS-mutant colorectal cancer. J Med Chem.

[86] Imyanitov EN, Levchenko EV, Kuligina ES, Orlov SV (2020). Treating non-small cell lung cancer with selumetinib: an up-to-date drug evaluation. Expert Opin Pharmacother.

[87] Flemington V, Davies EJ, Robinson D, Sandin LC, Delpuech O, Zhang P (2021). AZD0364 is a potent and selective ERK1/2 inhibitor that enhances antitumor activity in KRAS-mutant tumor models when combined with the MEK inhibitor, selumetinib. Mol Cancer Ther.

[88] Juric D, Rodon J, Tabernero J, Janku F, Burris HA, Schellens JHM (2018). Phosphatidylinositol 3-kinase α-selective inhibition with alpelisib (BYL719) in PIK3CA-altered solid tumors: results from the first-in-human study. J Clin Oncol.

[89] Ku BM, Jho EH, Bae Y-H, Sun J-M, Ahn JS, Park K (2015). BYL719, a selective inhibitor of phosphoinositide 3-Kinase α, enhances the effect of selumetinib (AZD6244, ARRY-142886) in KRAS-mutant non-small cell lung cancer. Invest New Drugs.

[90] Zhang Z, Morstein J, Ecker AK, Guiley KZ, Shokat KM (2022). Chemoselective covalent modification of K-Ras(G12R) with a small molecule electrophile. J Am Chem Soc.

[91] Zhang Z, Guiley KZ, Shokat KM (2022). Chemical acylation of an acquired serine suppresses oncogenic signaling of K-Ras(G12S). Nat Chem Biol.

[92] Knox JE, Jiang J, Burnett GL, Liu Y, Weller CE, Wang Z (2022). Abstract 3596: RM-036, a first-in-class, orally-bioavailable, Tri-complex covalent KRASG12D(ON) inhibitor, drives profound anti-tumor activity in KRASG12D mutant tumor models. Cancer Res.

[93] Christensen JG, Hallin J (2022). The KRASG12D inhibitor MRTX1133 elucidates KRAS-mediated oncogenesis. Nat Med.

[94] Mao Z, Xiao H, Shen P, Yang Y, Xue J, Yang Y (2022). KRAS(G12D) can be targeted by potent inhibitors via formation of salt bridge. Cell Discov.

[95] Kessler D, Gmachl M, Mantoulidis A, Martin LJ, Zoephel A, Mayer M (2019). Drugging an undruggable pocket on KRAS. Proc Natl Acad Sci USA.

[96] Tran TH, Alexander P, Dharmaiah S, Agamasu C, Nissley DV, McCormick F (2020). The small molecule BI-2852 induces a nonfunctional dimer of KRAS. Proc Natl Acad Sci USA.

[97] Titze-de-Almeida R, David C, Titze-de-Almeida SS (2017). The race of 10 synthetic RNAi-based drugs to the pharmaceutical market. Pharm Res.

[98] Kamerkar S, LeBleu VS, Sugimoto H, Yang S, Ruivo CF, Melo SA (2017). Exosomes facilitate therapeutic targeting of oncogenic KRAS in pancreatic cancer. Nature.

[99] Zhao X, Liu L, Lang J, Cheng K, Wang Y, Li X (2018). A CRISPR-Cas13a system for efficient and specific therapeutic targeting of mutant KRAS for pancreatic cancer treatment. Cancer Lett.

[100] Lu Y, Xue J, Deng T, Zhou X, Yu K, Deng L (2020). Safety and feasibility of CRISPR-edited T cells in patients with refractory non-small-cell lung cancer. Nat Med.

[101] Li X, Pu W, Zheng Q, Ai M, Chen S, Peng Y (2022). Proteolysis-targeting chimeras (PROTACs) in cancer therapy. Mol Cancer.

[102] Escher TE, Satchell KJF (2023). RAS degraders: the new frontier for RAS driven cancers. Mol Ther.

[103] Bond MJ, Chu L, Nalawansha DA, Li K, Crews CM (2020). Targeted degradation of oncogenic KRASG12C by VHL-recruiting PROTACs. ACS Cent Sci.

[104] Li L, Wu Y, Yang Z, Xu C, Zhao H, Liu J (2021). Discovery of KRas G12C-IN-3 and pomalidomide-based PROTACs as degraders of endogenous KRAS G12C with potent anticancer activity. Bioorg Chem.

[105] Nussinov R, Tsai CJ, Jang H (2021). Anticancer drug resistance: an update and perspective. Drug Resist Updat.

[106] Gurreri E, Genovese G, Perelli L, Agostini A, Piro G, Carbone C (2023). KRAS-dependency in pancreatic ductal adenocarcinoma: mechanisms of escaping in resistance to KRAS inhibitors and perspectives of therapy. Int J Mol Sci.

[107] Khleif SN, Abrams SI, Hamilton JM, Bergmann-Leitner E, Chen A, Bastian A (1999). A phase I vaccine trial with peptides reflecting ras oncogene mutations of solid tumors. J Immunother.

[108] Palmer DH, Valle JW, Ma YT, Faluyi O, Neoptolemos JP, Jensen Gjertsen T (2020). TG01/GM-CSF and adjuvant gemcitabine in patients with resected RAS-mutant adenocarcinoma of the pancreas (CT TG01-01): a single-arm, phase 1/2 trial. Br J Cancer.

[109] Quandt J, Schlude C, Bartoschek M, Will R, Cid-Arregui A, Schölch S (2018). Long-peptide vaccination with driver gene mutations in p53 and Kras induces cancer mutation-specific effector as well as regulatory T cell responses. Oncoimmunology.

[110] Delsing DJ, Offerman EH, van Duyvenvoorde W, van Der Boom H, de Wit EC, Gijbels MJ (2001). Acyl-CoA:cholesterol acyltransferase inhibitor avasimibe reduces atherosclerosis in addition to its cholesterol-lowering effect in ApoE*3-Leiden mice. Circulation.

[111] Zhu Y, Gu L, Lin X, Zhang J, Tang Y, Zhou X (2022). Ceramide-mediated gut dysbiosis enhances cholesterol esterification and promotes colorectal tumorigenesis in mice. JCI Insight.

[112] Liu J-Y, Fu W-Q, Zheng X-J, Li W, Ren L-W, Wang J-H (2021). Avasimibe exerts anticancer effects on human glioblastoma cells via inducing cell apoptosis and cell cycle arrest. Acta Pharmacol Sin.

[113] Zhu Y, Gu L, Lin X, Zhou X, Lu B, Liu C (2022). P53 deficiency affects cholesterol esterification to exacerbate hepatocarcinogenesis. Hepatology.

[114] Yang W, Bai Y, Xiong Y, Zhang J, Chen S, Zheng X (2016). Potentiating the antitumour response of CD8(+) T cells by modulating cholesterol metabolism. Nature.

[115] Pan J, Zhang Q, Palen K, Wang L, Qiao L, Johnson B (2019). Potentiation of Kras peptide cancer vaccine by avasimibe, a cholesterol modulator. EBioMedicine.

[116] Arbelaez CA, Estrada J, Gessner MA, Glaus C, Morales AB, Mohn D (2020). A nanoparticle vaccine that targets neoantigen peptides to lymphoid tissues elicits robust antitumor T cell responses. NPJ Vaccines.

[117] Berzofsky JA, Terabe M, Trepel JB, Pastan I, Stroncek DF, Morris JC (2018). Cancer vaccine strategies: translation from mice to human clinical trials. Cancer Immunol Immunother.

[118] Chaft JE, Litvak A, Arcila ME, Patel P, D'Angelo SP, Krug LM (2014). Phase II study of the GI-4000 KRAS vaccine after curative therapy in patients with stage I-III lung adenocarcinoma harboring a KRAS G12C, G12D, or G12V mutation. Clin Lung Cancer.

[119] Cohn A, Morse MA, O'Neil B, Whiting S, Coeshott C, Ferraro J (2018). Whole recombinant *Saccharomyces cerevisiae* yeast expressing Ras mutations as treatment for patients with solid tumors bearing Ras mutations: results from a phase 1 trial. J Immunother.

[120] Konduri V, Li D, Halpert MM, Liang D, Liang Z, Chen Y (2016). Chemo-immunotherapy mediates durable cure of orthotopic KrasG12D/p53−/− pancreatic ductal adenocarcinoma. Oncoimmunology.

[121] Keenan BP, Saenger Y, Kafrouni MI, Leubner A, Lauer P, Maitra A (2014). A Listeria vaccine and depletion of T-regulatory cells activate immunity against early stage pancreatic intraepithelial neoplasms and prolong survival of mice. Gastroenterology.

[122] Chaudhary N, Weissman D, Whitehead KA (2021). mRNA vaccines for infectious diseases: principles, delivery and clinical translation. Nat Rev Drug Discov.

[123] Miao L, Zhang Y, Huang L (2021). mRNA vaccine for cancer immunotherapy. Mol Cancer.

[124] Lorentzen CL, Haanen JB, Met Ö, Svane IM (2022). Clinical advances and ongoing trials on mRNA vaccines for cancer treatment. Lancet Oncol.

[125] Tran E, Robbins PF, Lu Y-C, Prickett TD, Gartner JJ, Jia L (2016). T-cell transfer therapy targeting mutant KRAS in cancer. N Engl J Med.

[126] Sim MJW, Lu J, Spencer M, Hopkins F, Tran E, Rosenberg SA (2020). High-affinity oligoclonal TCRs define effective adoptive T cell therapy targeting mutant KRAS-G12D. Proc Natl Acad Sci USA.

[127] Hong M, Clubb JD, Chen YY (2020). Engineering CAR-T cells for next-generation cancer therapy. Cancer Cell.

[128] Wagner J, Wickman E, DeRenzo C, Gottschalk S (2020). CAR T cell therapy for solid tumors: bright future or dark reality?. Mol Ther.

[129] Chen W, Ma Y, Shen Z, Chen H, Ma R, Yan D (2021). Humanized anti-CD19 CAR-T cell therapy and sequential allogeneic hematopoietic stem cell transplantation achieved long-term survival in refractory and relapsed B lymphocytic leukemia: a retrospective study of car-t cell therapy. Front Immunol.

[130] Zhao Y-L, Liu D-Y, Sun R-J, Zhang J-P, Zhou J-R, Wei Z-J (2021). Integrating CAR T-cell therapy and transplantation: comparisons of safety and long-term efficacy of allogeneic hematopoietic stem cell transplantation after CAR T-cell or chemotherapy-based complete remission in B-cell acute lymphoblastic leukemia. Front Immunol.

[131] Peng M, Mo Y, Wang Y, Wu P, Zhang Y, Xiong F (2019). Neoantigen vaccine: an emerging tumor immunotherapy. Mol Cancer.

[132] Parkhurst MR, Yang JC, Langan RC, Dudley ME, Nathan D-AN, Feldman SA (2011). T cells targeting carcinoembryonic antigen can mediate regression of metastatic colorectal cancer but induce severe transient colitis. Mol Ther.

[133] Chmielewski M, Hahn O, Rappl G, Nowak M, Schmidt-Wolf IH, Hombach AA (2012). T cells that target carcinoembryonic antigen eradicate orthotopic pancreatic carcinomas without inducing autoimmune colitis in mice. Gastroenterology.

[134] Klampatsa A, Dimou V, Albelda SM (2021). Mesothelin-targeted CAR-T cell therapy for solid tumors. Expert Opin Biol Ther.

[135] Morello A, Sadelain M, Adusumilli PS (2016). Mesothelin-targeted CARs: driving T cells to solid tumors. Cancer Discov.

[136] Watanabe K, Luo Y, Da T, Guedan S, Ruella M, Scholler J (2018). Pancreatic cancer therapy with combined mesothelin-redirected chimeric antigen receptor T cells and cytokine-armed oncolytic adenoviruses. JCI Insight.

